# RETRACTION: Analysis of Risk Factors of Infection in Diabetic Foot Patients

**DOI:** 10.1111/iwj.70671

**Published:** 2025-04-23

## Abstract

**RETRACTION:** HsuL. , LiL. , and PoonL. Y. , “Analysis of Risk Factors of Infection in Diabetic Foot Patients,” International Wound Journal 21, no.1 (2024): e14411, 10.1111/iwj.14411.37731215 PMC10788463

The above article, published online on 20 September 2023, in Wiley Online Library (http://onlinelibrary.wiley.com/), has been retracted by agreement between the journal Editor in Chief, Professor Keith Harding; and John Wiley & Sons Ltd. Following an investigation by the publisher, all parties have concluded that this article was accepted solely on the basis of a compromised peer review process. The editors have therefore decided to retract the article. The authors did not indicate their agreement with the retraction.

**RETRACTION:** L. Hsu , L. Li , and L. Y. Poon , “Analysis of Risk Factors of Infection in Diabetic Foot Patients,” International Wound Journal 21, no.1 (2024): e14411, 10.1111/iwj.14411.37731215 PMC10788463

The above article, published online on 20 September 2023, in Wiley Online Library (http://onlinelibrary.wiley.com/), has been retracted by agreement between the journal Editor in Chief, Professor Keith Harding; and John Wiley & Sons Ltd. Following an investigation by the publisher, all parties have concluded that this article was accepted solely on the basis of a compromised peer review process. The editors have therefore decided to retract the article. The authors did not indicate their agreement with the retraction.

